# Iatrogenic colorectal perforation caused by a clip

**DOI:** 10.1055/a-2163-2290

**Published:** 2023-10-06

**Authors:** Hirotaka Oura, Yasuki Hatayama, Erika Nomura, Harutoshi Sugiyama, Daisuke Murakami, Makoto Arai, Takayoshi Nishino

**Affiliations:** Department of Gastroenterology, Tokyo Women's Medical University Yachiyo Medical Center, Chiba, Japan

An iatrogenic colonic perforation (ICP) is a significant incident associated with colonoscopy, with recent guidelines detailing a treatment-related incidence of 0.02 %–8 % 1 . Although clips are frequently used to suture perforations 2 , ICPs caused by the clips themselves have not previously been reported. Here, we describe a case of an ICP occurring during endoscopic treatment that was caused by a clip in a colonic diverticulum.

A man aged in his eighties was referred to our hospital for treatment of colonic polyps. Colonoscopy revealed multiple colonic diverticula in the sigmoid colon. A trainee with less than 1 year of experience had attempted to use a clip to close the ulcer after endoscopic mucosal resection of a 5-mm Is polyp in the patient’s sigmoid colon. During the procedure, the scope was frequently pulled out toward the anal side owing to enhanced peristaltic spasm of the colon. During reinsertion with the clip opened, a laceration occurred in the mucosa because the metal part of the tip of the clip got caught in a small depression of a colonic flexure ( Fig. 1 a ; Video 1 ). No muscular layer was observed in the mucosal defect, consistent with a diagnosis of perforation at the diverticular site ( Fig. 1 b ). The wound was completely closed with clips ( Fig. 1 c ). A computed tomography (CT) scan of the abdomen taken after the examination showed air leakage outside of the colon ( Fig. 2 ). The patient was discharged after 1 week of conservative treatment.

**Fig. 1:**
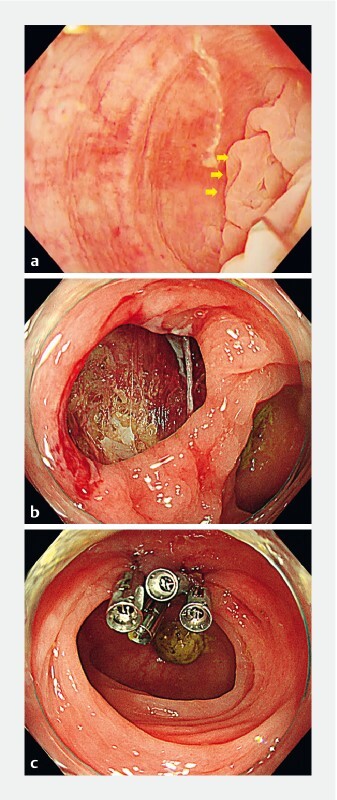
Endoscopic images showing: **a** a small depression in the mucosa (yellow arrows) that appears to be a diverticulum in the sigmoid colon where the tip of the clip got caught; **b** a perforation in the diverticular area, which lacked a muscle layer; **c** the perforation closed completely with four clips.

**Video 1** Iatrogenic colorectal perforation at a diverticulum caused by a clip that was being placed following endoscopic mucosal resection performed by a trainee.


**Fig. 2:**
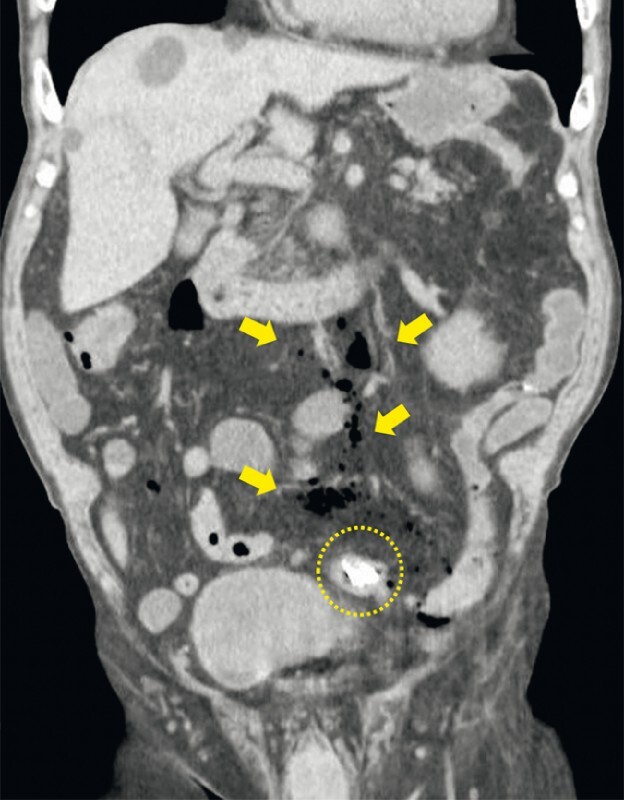
Image from a computed tomography scan performed immediately after the colonoscopy showing the clip used to suture the perforation of the sigmoid colon (dotted yellow line) and air leakage outside of the colon (yellow arrows).

We report a case of ICP at a colonic diverticulum caused by a metal clip tip. As has been previously reported in endoscopic treatment of tumors involving diverticula, diverticula lack or have a thin muscle layer 3 4 . Devices such as clips should be retracted into the forceps channel of the endoscope or the attachment hood before their insertion into the flexure of the colon, especially in patients with diverticula.

Endoscopy_UCTN_Code_CPL_1AJ_2AG

## Notes

Endoscopy E-Videos

https://eref.thieme.de/e-videos
*E-Videos* is an open access online section of the journal *Endoscopy* , reporting on interesting cases and new techniques in gastroenterological endoscopy. All papers include a high-quality video and are published with a Creative Commons CC-BY license. Endoscopy E-Videos qualify for HINARI discounts and waivers and eligibility is automatically checked during the submission process. We grant 100% waivers to articles whose corresponding authors are based in Group A countries and 50% waivers to those who are based in Group B countries as classified by Research4Life (see: https://www.research4life.org/access/eligibility/ ). This section has its own submission website at https://mc.manuscriptcentral.com/e-videos
